# Influence of infant feeding practices on the occurrence of malnutrition, malaria and anaemia in children ≤5 years in the Mount Cameroon area: A cross sectional study

**DOI:** 10.1371/journal.pone.0219386

**Published:** 2019-07-18

**Authors:** Gillian Nkeudem Asoba, Irene Ule Ngole Sumbele, Judith Kuoh Anchang-Kimbi, Samuel Metuge, Rene Ning Teh

**Affiliations:** 1 Department of Social Economy and Family Management, Higher Technical Teachers' Training College, University of Buea, Kumba, Cameroon; 2 Department of Zoology and Animal Physiology, University of Buea, Buea, Cameroon; Amala Institute of Medical Sciences, INDIA

## Abstract

**Background:**

The objective of this study was to evaluate the influence of different infant feeding habits on the occurrence of malnutrition, *Plasmodium falciparum* parasitaemia and anaemia in children **≤**5 years in the Mount Cameroon area.

**Methodology:**

A total of 1227 children ≤5 years of age were recruited in a descriptive cross-sectional study. Socio demographic data and information on the different infant feeding habits was obtained by the use of semi-structured questionnaire. Nutritional status was assessed by the use of anthropometric measurements. *Plasmodium* was detected by light microscopy and haemoglobin was measured by use of an auto-haematology analyser. Anaemia as well as its severity was classified based on WHO standards. The associations between variables were assessed using logistic regression analysis.

**Results:**

The prevalence of exclusive breast feeding (EBF) was 22.6%, mixed feeding (MF) was 60.1% and those not breastfed (NBF) at all was 17.3%. The prevalence of malnutrition, *P*. *falciparum* parasitaemia and anaemia was 32.6%, 30.4% and 77.3% respectively. Children who had EBF had significantly lower (P <0.001) prevalence of malaria parasite (16.2%) than those NBF at all (61.3%). The prevalence of anaemia was significantly higher (P <0.001) in children who had MF (80.5%) while, severe and moderate anaemia was highest in those NBF at all (6.6%, 67.1% respectively; P = 0.029) than their counterparts. The significant predictors of anaemia were age group (P <0.001), marital status (P <0.001) and educational level of parent (P <0.001), that for malaria parasitaemia was infant feeding habit (MF: P< 0.001 and NBF: P <0.001) and malnutrition was age group (≤2 years: P <0.008 and 2.1–4.0 years: P = 0.028).

**Conclusion:**

The infant feeding habit significantly influenced the occurrence of malaria parasite infection and not malnutrition and anaemia, hence EBF should be encouraged in malaria endemic zones.

## Background

Infant feeding methods is a major determinant of children’s nutritional status [1, 2]. Breastfeeding has been associated with reduction in morbidity and mortality in children less than 5 years, particularly those exclusively breastfed up to 4 and 6 months of age [3, 4]. The world health organisation (WHO) recommends exclusive breastfeeding of all infants until six months of age [5]. In spite of all the sensitization, the prevalence of exclusive breastfeeding remains low [6,7].

Malnutrition is one of the principal underlying causes of death for many of the world’s children, contributing to more than a third of under-five deaths globally. About 178 million children globally are stunted and Africa has the highest rates [8]. Generally, the risk of malnutrition in the first 2 years of life has been directly linked with poor breastfeeding and complementary feeding practices of mothers together with high rates of infectious diseases [9]. Addressing the influence of complementary feeding practice of mothers on the nutritional status of children may be an important approach towards reducing the burden of child malnutrition and other infectious diseases such as malaria.

Globally, malaria is still a public health concern with approximately 445,000 malaria related deaths occurring in 2016 and Cameroon alone accounted for 3% of this number [10]. As seen in many different areas of the globe, the malaria burden and transmission intensity in Cameroon is heterogeneous [11]. Although different control measures including free treatment of uncomplicated malaria in children under five years with artemisinin-based combination therapies (ACTs) and nation-wide distribution of long-lasting insecticidal net [12] have been implemented in Cameroon, the disease burden is still of concern [12–14]. Most of these deaths occurred in children under the age of 5 years, who lack naturally acquired immunity and as a consequence have the highest rates of infection, complications, and mortality [2]. However, despite lacking naturally acquired immunity, the first months of life are marked by a low incidence of malaria [15], which may be due to transplacental transfer of protective maternal immunoglobulin G (IgG) [16], although other studies do not support these findings [15].

Increased malaria prevalence among exclusively breastfed infants have been reported in Uganda and Malawi, whereas a study conducted in Nigeria stated the contrary [17, 18]. In spite of the different findings, all three studies suggested that EBF had no significant effect on malaria infection risk [17, 18]. However, the study from Nigeria was based on questionnaires and is subject to recall bias, whereas the Malawian study did not use the proper EBF definition as proposed by the WHO [18, 19].

Nutritional status and immune responses to infection are closely related. Akiyama *et al*. [20] reported a close tie between malnutrition particularly stunting, and *Plasmodium falciparum* malaria among children, with the under five years recording the highest burden. There have been a number of studies determining the prevalence of anaemia among infants and young children in Cameroon [12–14], however, examining a richer set of correlates such as infant feeding habits is essential to painting a more complete picture of the population most at risk of anaemia and hence of considerable policy relevance. Consequently, the objective of the study was to evaluate the influence of different types of infant feeding habits such as EBF, MF and no breast feeding (NBF) on the occurrence of malnutrition, *P. falciparum* parasitaemia and anaemia in children **≤**5years in the Mount Cameroon area.

## Methods

### Study area and participants

**Fig 1:**
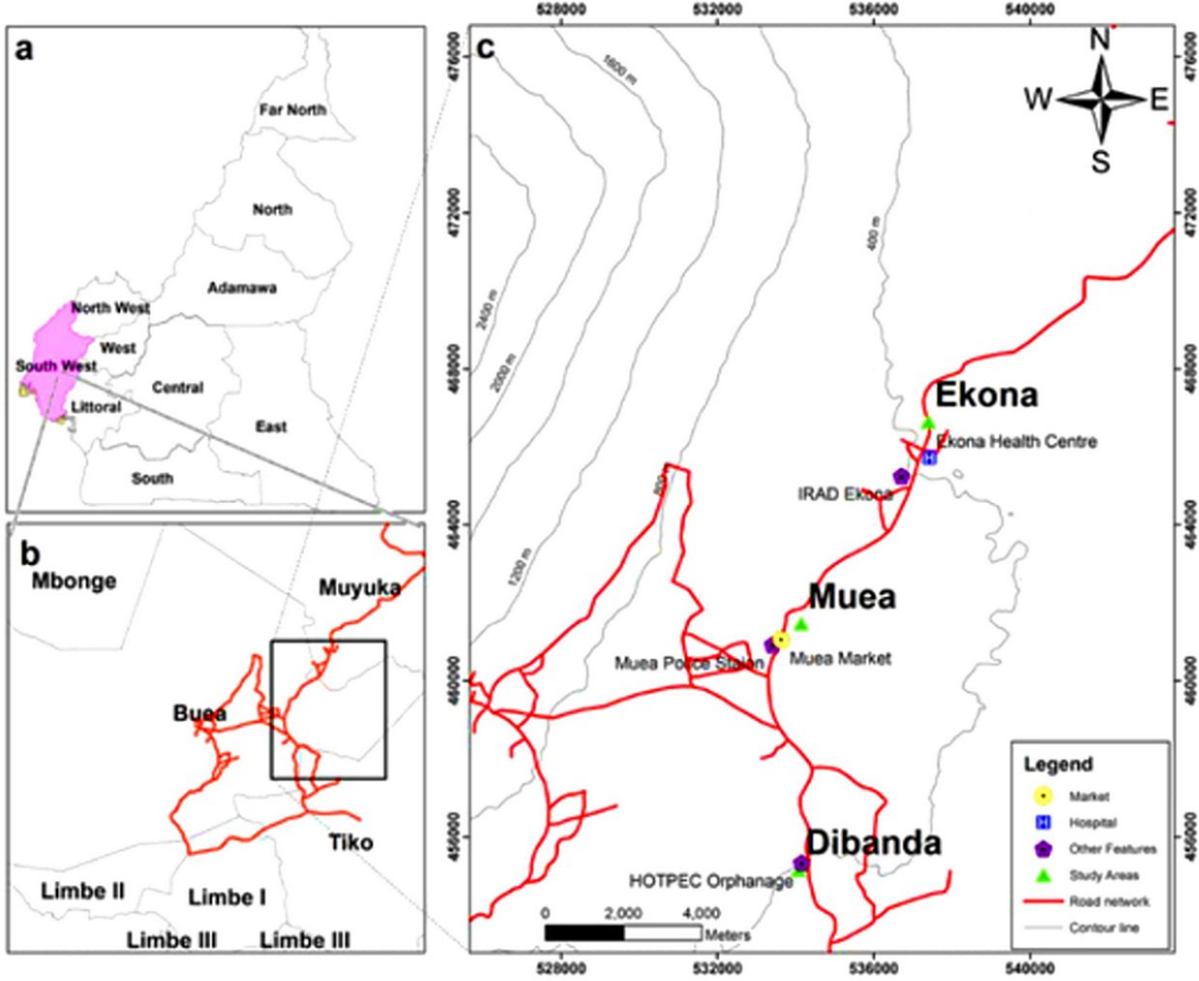
Map showing the different study areas.

This study was carried out in the semi-rural communities of Muea, Ekona and Dibanda, situated at the foot of Mount Cameroon in the South West Region of Cameroon as shown in Fig 1.

In all of the three study sites, the rainy season is characterized by frequent light showers that spans from March to October and the dry season runs from November to February. Muea is situated in the rain forest ecozone on the Eastern flank of the active volcanic Mount Cameroon. It is located 562m above sea level at longitude 09°18’53”E and latitude 04°05’14”N and has been described in detail by Sumbele *et al*. [21]. *Plasmodium falciparum* accounts for up to 96% of malaria infections in the area [22]. Dibanda is located on the windward side of the eastern slope of Mount Cameroon with an altitude of 530m above sea level, 25km from the Atlantic Ocean, at longitude 09°18’47”E and latitude 04°05’09” N. The area has been described in detail by Nyasa *et al*. [23]. Ekona is located 898m above sea level at longitude 9°20’ 4”E and latitude 4°14’0”N. The majority of inhabitants are involved in farming, trading or livestock keeping. The two seasons of Ekona have been described in details by Wandji *et al*. [24].

All children **≤**5 years resident in Muea, Ekona and Dibanda whose parents/caregivers consented to participate in the study were recruited. Children with malaria parasite presenting with fever, rigor and chills, joint pains, malaise, abdominal pain, nausea and vomiting were considered symptomatic and children with none of these symptoms were considered as asymptomatic. Children with severe malaria (cerebral malaria), severe complications of malnutrition including kwashiorkor; known underlying chronic or severe disease (e.g., HIV/AIDS, cardiac, renal or hepatic disease, sickle cell), presence of febrile conditions due to diseases other than malaria requiring hospitalization in intensive care or stabilization, congenital malformations and congenital disease which could alter the outcome of the study were excluded from the study.

### Study design

This observational cross-sectional study was carried out between the months of March and October, 2018 to include the rainy season (March-October) which has been reported as the peak malaria transmission period in the Mount Cameroon area [25]. After obtaining an ethical clearance, administrative and local authorizations from the chief and block heads in the neighbourhood, the study team proceeded to the field for sample collection.

### Sample size, method and sampling unit

The minimum number of samples required for the study was calculated using formula; n = z^2^_α/2_(1 –a) P (1—P)/d^2^ where n is the minimum sample size required; z is 1.96 which is the standard normal deviate; a is absolute precision at 5%; p is 66.2% which is the proportion of malaria prevalence [26] and d is 0.05 (5%) the required margin of error. This gave a minimum sample size of 327 participants for each locality. Considering a possible participation of more than one child per family, loss of samples due to blood clotting and incomplete data entry, the sample size was adjusted by approximately 10% to a minimum of 360.

A multistage cluster sampling method was used to obtain the required sample. In the first stage, 3 communities were randomly selected from the 29 rural communities in the Mount Cameroon area. In the second stage, 32 clusters were randomly selected within the three communities. Within each cluster, all the households were selected until the required sample size was attained. In a household with more than one child in this age group, only one was randomly selected. At the start of the study in each site, the parents and guardians of the children were educated on the study protocol and the benefits of participation highlighted at their various neighbourhoods. Ensuing administrative clearances and ethical approval for the study, informed consent/assent forms explaining the purpose, risks, and benefits of the study were given to parent/caregivers. Participants were invited to the data collection location in each community by their local chiefs and coordination was organised by the block heads of the various communities. Upon obtaining consent/assent from the participants, semi-structured questionnaires were administered to the mothers/caregivers to get socio-demographic data and information on the different feeding methods. Following administration of the questionnaire, body temperature and anthropometric measurements were obtained and blood sample was collected from each child for malaria parasite identification and a full blood count assessment.

### Questionnaire survey

Data was collected regarding the following: demographics (gender and age), feeding habits (exclusive breastfeeding and duration/ mixed feeding/no breastfeeding), types of local weaning foods, mother’s knowledge on balance diet, history of fever in the preceding 2–3 days, history of consumption of any medication in the preceding month, history of diarrhoea, mosquito bed net use, availability of toileting facilities in household, sources of household water, marital status of parents and parent/guardian educational level. Infants were classified as being exclusively breastfed using the definitions proposed by the WHO [2]. In addition, a child was said to have mixed feeding when he/she was given a combination of breast milk and local infant formulae before six months and the last category of children were those who were not given breast milk at all from birth and were fed with local infant formula.

### Clinical evaluation

The axillary temperature was measured using an electronic thermometer and fever was defined as temperature ≥37.5°C. Anthropometric measurements such as height and weight were measured using a measuring tape and a beam balance (Terraillon, Paris) while the ages of the children were obtained from their mothers/caregivers and or birth certificates. Under-nutrition indices such height-for age (HA), weight-for-age (WA), and weight-for-height (WH) standard deviation (SD) scores (Z scores) were computed based on the WHO growth reference curves using the WHO AnthroPlus for personal computers manual [27]. A child was identified as being undernourished if he or she scored <-2 SD in one of the anthropometric indices of HA (stunting), WA (underweight) and WH (wasting) indices, while corresponding Z scores of <-3 SD were considered indicative of severe under-nutrition [28]. A child with a standard deviation score of <-2 for any of the anthropometric indices was classified as malnourished.

### Laboratory methods

Approximately 2–3 mL of venous blood samples were collected using sterile syringes from enrolled children into labelled ethylenediaminetetraacetate (EDTA) tubes. Thick and thin blood films were prepared on the same slide and air-dried in the field. The remaining blood samples were transported on ice in a cool box to the Malaria Research Laboratory, University of Buea for a full blood count analysis. The blood films were stained with 10% Giemsa for 20 minutes after the thin smear had been fixed with absolute methanol. The films were examined following standard procedure for the detection and identification of malaria parasites [29]. Parasite density was expressed as asexual parasites per μL using the participant’s white blood cell (WBC) count. Parasitaemia was categorised as low (<1,000 parasites/μL of blood), moderate (1,000–4,999 parasites/ μL of blood), high (5,000–99,999 parasites/ μL of blood), and hyperparasitaemia (≥100,000 μL of blood) [12].

An auto-haematology analyser (MINRAY 2800 BC) was used to assess haematological parameters following the manufacturer’s instructions. The haemoglobin (Hb) measured was used to define the status of anaemia. The condition of anaemia was defined as Hb <11.0 g/dL [29] and further categorized as severe (Hb <7.0 g/dL), moderate (Hb between 7.0 and 10.0 g/dL), and mild (Hb between 10.1 and <11 g/dL) [29].

### Statistical analysis

Data collected was cleaned up and analysed using the IBM-Statistical Package for Social Sciences (IBM-SPSS) version 20. Continuous variables were summarized into means and standard deviations (SD) and categorical variables reported as frequencies and percentages were used to evaluate the descriptive statistics. The differences in proportions were evaluated using Pearson’s Chi-Square (χ^2^). Group means were compared using analysis of variance (ANOVA), Student’s t-test, Mann-Whitney U test and Kruskal W0061llis test where appropriate. Parasite density was log-transformed before analysis. Multi-collinearity test was performed and any covariate with a P value <0.2 in the bivariate analysis was included in the multivariable logistic model. Significant levels were measured at 95% confidence interval (CI) with significant differences set at <0.05.

### Ethics statement

The study was approved by the Institutional Review Board hosted by the Faculty of Health Sciences, University of Buea (2018/004/UB/FHS /IRB) following administrative clearance from the South West Regional Delegation of Public Health, Cameroon. Informed consent/assent forms were given or read and explained to parents or caregivers of the children at presentation. The purpose and benefits of the study as well as the amount of blood to be collected from each child was clearly stated on the consent/assent forms. Only participants who gave written and/or verbal consent or assent took part in the study. Participation was strictly voluntary and parents or caregivers were free at any point in time to stop the participation of the child/children in the study. All cases of malaria and those with moderate to severe anaemia as well malnutrition were referred to the nearest health centre for appropriate treatment and follow up.

## Results

### Characteristics of study participants

The socio-demographic and clinical characteristics of the study participants are shown in Table 1. A total of 1227 children with a mean (SD) age of 3.1 (1.6) years, residing in Ekona (35.5%, 436), Dibanda (31.9%, 391) and Muea (32.6% 400) in the Mount Cameroon area were evaluated. The females were more (52.2%) than the males (47.8%) although not significant. Participation within age groups was comparable with more of the children in the ≤2 years (34.9%) age group. A greater proportion of the parents/guardians of the children (37.1%) had no formal education. The prevalence of malaria parasite, anaemia and malnutrition in children ≤5 years was 30.4%, 77.3% and 32.6% respectively. The most common form of malnutrition was stunting (27.1%).

**Table 1:** Socio-demographic and clinical characteristics of study participants.

Parameter	Category	% (n)
Study sites	Ekona (n)	35.5 (436)
	Dibanda (n)	31.9 (391)
	Muea (n)	32.6 (400)
Sex	Female	52.2 (641)
	Male	47.8 (586)
Age groups in years	≤2	34.9 (428)
	2.1–4.0	35.1 (431)
	4.1–5	30.0 (368).
Educational level of parent/caregiver	No formal	37.1 (444)
	Primary	26.8 (320)
	Secondary (n)	27.3 (326)
	Tertiary (n)	8.9 (106)
Clinical	Mean age (SD) in years	3.1 (1.6)
	Mean weight (SD) in kg	14.39 (5.1)
	Mean height (SD) in cm	92.0 (18.2)
	Mean temperature (SD) in °C	36.5 (0.6)
	Fever prevalence	6.6 (81)
	Malaria parasite prevalence	30.4 (373)
	Mean haemoglobin (SD) level (g/dL)	9.9 (1.5)
	Anaemia prevalence	77.3 (948)
	Malarial anaemia prevalence	23.5 (288)
	Non malaria anaemia prevalence	53.8 (660)
	Prevalence of malnutrition	32.6 (400)
	Prevalence of wasting	6.1 (75)
	Prevalence of underweight	10.4 (127)
	Prevalence of stunting	27.1 (333)

### Feeding practices in the study population

The overall proportion of children who had EBF, MF and NBF from the 1227 children was 22.6%, 60.1% and 17.3% respectively. For children who had MF before 6 months, local weaning food namely: Vita Force (VT), Soytine (ST), Soyaconia (SC), Soya Pap (SP), TantyReine (TR), Dina Baby (DB) and standard of Cerelac milk in few cases were obtained from local markets, and given to the children alongside with breast milk. Meanwhile only some of these local weaning foods and standard of Cerelac milk were given to the 17.3% of children who were not breast fed at all from birth. The macronutrient composition, energy level, moisture content and micronutrient compositions of these local weaning foods were within the FAO recommendations for children as revealed in a previous study by the authors [30].

As shown in Table 2, the highest proportion of mothers who exclusively breast fed their children were from Muea (26.8%), followed by Dibanda (23.3%) and the least from Ekona (18.1%). Significantly (P = 0.004), most parents in the three study sites gave their children mixed feeding from birth. Among the 1196 children whose parents gave information on their educational level, a statistically significant (P <0.001) proportion of parents with tertiary education exclusively breastfed their children (34.0%) while, parents with no formal education had the highest proportion of children who were given mixed feeding (64.2%) and those not breast-fed (20.5%).

**Table 2:** Association between the study sites and educational level on different infant feeding methods.

Parameter		No. examined	Infant feeding methods, % (n)			χ2
			EBF	Mixed feeding	No breast milk	P- value
Study site	Ekona	437	18.1 (79)	66.6 (291)	15.3 (67)	
	Dibanda	390	23.3 (91)	59.7 (233)	16.9 (66)	15.469
	Muea	400	26.8 (107)	53.4 (214)	19.8 (79)	0.004
	Total	1227	22.6 (277)	60.1 (738)	17.3 (212)	
*Educational level of parent/caregiver	No formal	444	15.3 (68)	64.2 (285)	20.5 (91)	
	Primary	320	25.6 (82)	60.0 (192)	14.4 (46)	28.547
	Secondary	326	26.7 (87)	56.7 (185)	16.6 (54)	0.001
	Tertiary	106	34.0 (36)	48.1 (51)	17.9 (19)	
	Total.	1196	22.8 (273)	59.6 (713)	17.6 (210)	

* Information obtained from 1196 parents/ caregivers only.

### Age, nutritional status and infant feeding practice

**Fig 2:**
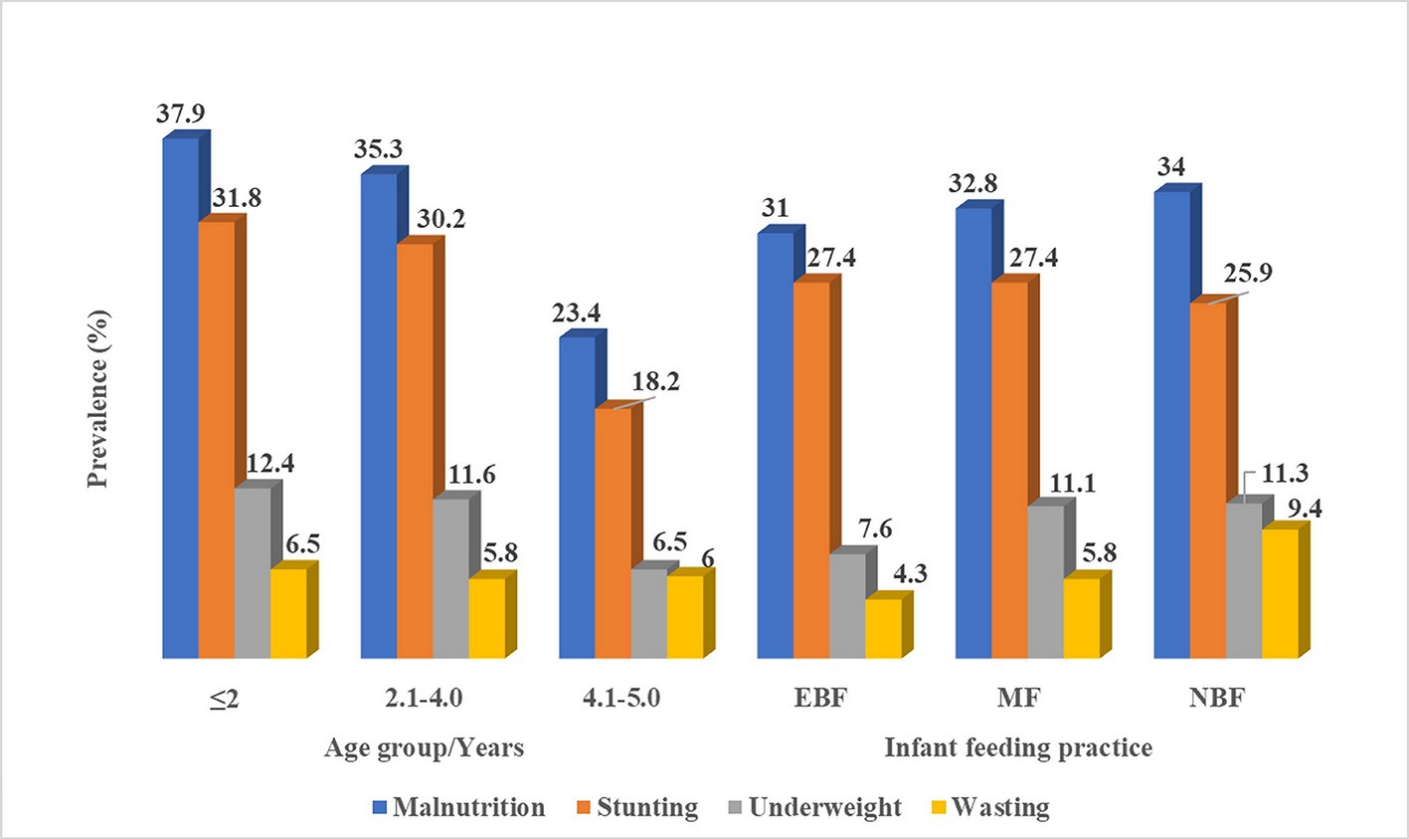
Effect of age and infant feeding practice on the prevalence of malnutrition and its forms.

The prevalence of malnutrition and its forms (stunting and underweight) decreased significantly (P <0.001, P <0.001 and P = 0.015 respectively) with the age group. The highest prevalence of malnutrition was observed in the ≤2 years age group (37.9%) and least in 4.1–5.0 years (23.4%). Similarly, the prevalence of stunting (31.8%) and underweight (12.4%) was highest among the ≤2 years age group. Although the prevalence of wasting was highest among the ≤ 2 years age group (6.5%) than contemporaries, the difference was not significant as shown in Fig 2.

Although not statistically significant, children who were not breastfed at all had the highest prevalence of malnutrition (34.0%), followed by those with MF habits (32.8%) and the least occurred in children who were exclusively breast fed (31.0%). Across the different forms of malnutrition, children who were not breastfed at all had the highest prevalence of wasting (9.4%) that approached significance (P = 0.058) and underweight (11.3%) when compared with their counterparts (Fig 2).

### Malaria parasite and infant feeding habits

The prevalence of malaria parasite was comparable between sites (P = 0.398), gender (P = 0.79) and age groups (P = 0.726). On the other hand, a significant association was observed between infant feeding habits and the malaria parasite where, children who had EBF had significantly lower (P <0.001) prevalence of malaria parasite (16.2%) when compared with MF (26.8%) and those NBF at all (61.3%) as shown in Table 3.

**Table 3:** Malaria parasite prevalence and density with respect to study site, sex, age and infant feeding habits.

Parameter	Category	No. examined	Prevalence of malaria parasite (n)	P value	GMPD (Range)/ μL of blood	P- value
Site	Ekona	436	32.7 (143)	0.398	1222 (60–29000)	< 0.001^a^*
	Dibanda	391	29.2 (144)		222 (90–1120)	
	Muea	400	29 (116)		458 (54–31500)	
Gender	Male	586	30.0 (176)	0.79	579 (60–29000)	0.245
	Female	641	30.7 (197)		498 (54–31500)	
Age group in years	≤ 2	428	29.2 (125)	0.731	701 (62–31500)	< 0.001^b^*
	2.1–4	431	30.4 (131)		556 (60–20280)	
	4.1–5	368	31.8 (117)		371 (535–29000)	
Infant Feeding habit	EBF	277	16.2 (45)		619 (66–31500)	0.48
	MF	738	26.8 (198)	<0.001*	554 (54–29000)	
	NBF	212	61.3 (130)		481 (75–20280)	

*Statistically significant P value.

^a^ Difference in GMPD in the different study sites and sex determined by Mann Whitney *U* test.

^b^ Difference in GMPD in the different age groups determined by Kruskal-Wallis test.

The geometric mean parasite density (GMPD)/μL of blood was highest in children of Ekona (1222 parasites/ μL of blood) than the other sites (P <0.001) and in children ≤2 years (701 parasites/ μL of blood) than their contemporaries (P <0.001). Also, although not statistically significant, the GMPD was highest in males (579 parasites/ μL of blood) and in children who had EBF (619 parasites/ μL of blood) than their respective counterparts (Table 3).

**Fig 3:**
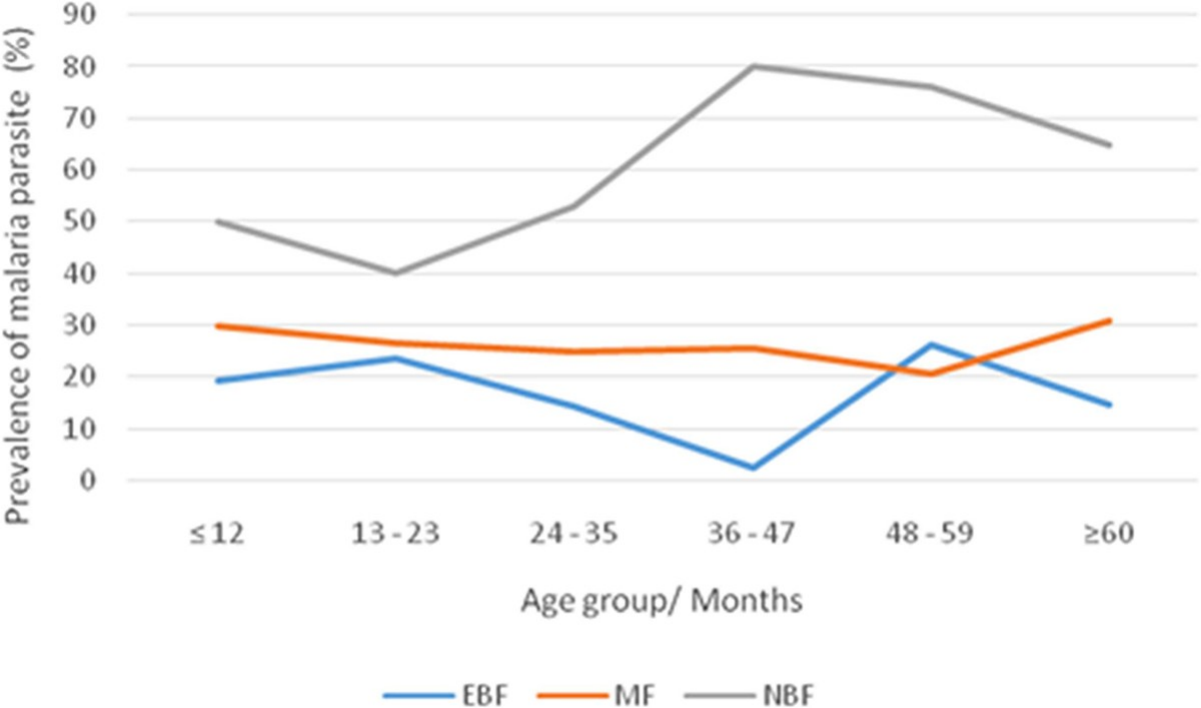
Prevalence of malaria parasites in different age groups among children with different feeding habits.

In addition, children that were EBF had the lowest malaria parasite prevalence across all ages with a peak increase between 48 and 59 months. Conversely, children who were not breast fed had the highest malaria prevalence across all ages with a steady drop from the 36^th^ month as revealed in Fig 3.

### Anaemia prevalence and severity

The prevalence of anaemia was significantly higher in males (80.7%), children ≤2 years of age (86.7%) and those who had MF (80.5%) when compared with members of their respective clusters as shown in Table 4. Relating to the severity of anaemia, the prevalence of severe and moderate anaemia was highest in males (5.7%, 60.3%), children ≤2 years of age (6.2%, 61.7%) and those who had NBF (6.6%, 67.1%). The differences were statistically significant at P = 0.049, P <0.001 and P = 0.029 respectively as shown in Table 4.

**Table 4:** Effect of study site, gender, age and infant feeding practice on anaemia prevalence and severity.

Parameter		No. examined	Prevalence of anaemia(n)	Anaemia severity		
				Severe % (n)	Moderate % (n)	Mild % (n)
Study site	Ekona	437	78.3 (342)	6.1 (21)	57.6 (197)	36.3 (124)
	Dibanda	390	80.0 (312)	4.5 (14)	58.3 (182)	37.2 (116)
	Muea	400	73.5 (294)	2.4 (7)	60.2 (177)	37.4 (110)
Test statistic	χ2		5.135		5.303	
	P-value		0.077		0.258	
Gender	Male	586	80.7 (473)	5.7 (27)	60.3 (285)	34.0 (161)
	Female	641	74.1 (475)	3.2 (15)	57.1 (271)	39.8 (189)
Test statistic	χ2		7.622,		6.017	
	P-value		**0.006**		**0.049**	
Age group in years	≤ 2	428	86.7 (371)	6.2 (23)	61.7 (229)	32.1 (119)
	2.1–4.0	431	75.4 (325)	4.9 (16)	60.3 (196)	34.8 (113)
	4.1–5.0	368	68.5 (252)	1.2 (3)	52.0 (131)	46.8 (118)
Test statistic	χ2		38.626,		20.892	
	P-value		**< 0.001**		**< 0.001**	
Infant feeding practice	EBF	277	67.5 (187)	3.7 (7)	56.7 (106)	39.6 (74)
	MF	738	80.5 (594)	4.0 (24)	56.9 (338)	39.1 (232)
	NBF	212	78.8 (167)	6.6 (11)	67.1 (112)	26.3 (44)
Test statistic	χ2		19.645		10.753	
	P-value		**0.001**		**0.029**	

P-values in bold are statistically significant

### Predictors for anaemia, malaria parasitaemia and malnutrition

The logistic regression model with age, gender, level of education and marital status of guardian, malaria status, infant feeding methods as well as nutritional status as the independent variables revealed age group (P <0.001), marital status (P <0.001) and educational level of parent (P <0.001) as significant predictors of anaemia as shown in Table 5. Children ≤2 years of age and single parents were 2.25 and 6 times respectively, more likely to be anaemic than their counterparts. In addition, children from guardians with no formal, primary and secondary level of education were 4, 34 and 88 times respectively more likely to be anaemic than children from guardian with tertiary level of education. The logistic regression model with malaria status as dependent variable, revealed the infant feeding habit as a significant predictor of malaria parasitaemia. Children who had MF (P <0.001) and those NBF (P <0.001) were 2 and 8 times respectively more likely to have malaria parasitaemia than children that had EBF. With respect to malnutrition as the dependent variable, age was the only significant predictor. Children ≤2 years and those between 2.1 and 4.0 years, were 1.73 and 1.59 times respectively more likely to be undernourished than children between 4.1 and 5.0 years old.

**Table 5:** Logistic regression model examining factors influencing prevalence of anaemia, malaria parasite and malnutrition in children.

Variables	Anaemia				Malaria parasitaemia				Malnutrition			
	Bivariate		Multivariate		Bivariate		Multivariate		Bivariate		Multivariate	
	COR	P value	COR	P value	COR	P value	AOR	P Value	COR	P value	COR	P value
Age group (Years)												
4.1–5.0	Reference		Reference		1.13	0.423	-	-	Reference		Reference	
2.1–4.0	1.41	**0.03**	0.03	0.569	1.06	0.703	-	-	1.79	**<0.001**	1.73	**0.008**
≤ 2	3.00	**<0.001**	0.88	**0.001**	Reference		Reference		2.00	**<0.001**	1.59	**0.028**
Gender												
Female	Reference		Reference		1.03	0.790	-	-	Reference		Reference	
Male	1.46	**0.006**	1.19	0.371	Reference		Reference		1.08	0.545	-	-
Marital status												
Married	Reference		Reference		Reference		Reference		Reference		Reference	
Single	11.9	**<0.001**	6.08	**<0.001**	1.18	0.253	-	-	1.33	0.051	1.10	0.669
Educational level of parents												
Tertiary	Reference		Reference		Reference		Reference		-	-	-	-
Secondary	4.37	**<0.001**	3.98	**<0.001**	1.22	0.418	-	-	-	-	-	-
Primary	38.31	**<0.001**	33.77	**<0.001**	0.98	0.923	-	-	-	-	-	-
No formal	151	**<0.001**	88.22	**<0.001**	1.20	0.437	-	-	-	-	-	-
Infant feeding habit												
EBF	Reference		Reference		Reference		Reference		Reference		Reference	
MF	1.99	**<0.001**	1.46	0.088	1.89	**<0.001**	1.90	**<0.001**	1.08	0.60	-	-
NBF	1.78	**0.006**	1.56	0.098	8.17	**<0.001**	8.26	**<0.001**	1.14	0.49	-	-
Malaria status												
Negative	Reference		Reference		-	-	-	-	1.19	0.204	1.20	0.303
Positive	1.0	0.978	-	-	-	-	-	-	Reference		Reference	
Anaemic Status												
No	-	-	-	-	Reference		-	-	Reference		Reference	
Yes	-	-	-	-	1.0	0.978	-	-	1.39	**0.03**	1.22	0.385
Undernourished												
No	Reference		Reference		1.19	0.204	1.24	0.123	-	-	-	-
Yes	1.39	**0.03**	1.12	0.713	Reference		Reference		-	-	-	-

P values in bold are statistically significant.

## Discussion

This cross-sectional study examines the influence of feeding practices on the occurrence of malnutrition, malaria and anaemia in children ≤5 years in the Mount Cameroon Area. The rate of exclusive breast feeding in this area (22.6%) is lower than the 28.2% reported by the World Bank in Cameroon in 2014 [2]. The lower rate could be attributed to the fact that most of the study participants had not gotten any form of formal education hence, were not very aware of the important role feeding a child exclusively on breast milk in the first six months of life had to do with the building of the child’s immune system and the fight against opportunistic infections. Furthermore, most of the participants in the study sites did not attend their antenatal classes during pregnancy and only went to the hospital to deliver.

Overall, 77.4% of the children in the study areas were introduced to complementary food before the recommended age of 6 months. This high frequency is similar with studies in Nasarawa, Nigeria, where 69–82% of children are reported to have been introduced to complementary foods before 6 months [31]. Early introduction to solid foods, early cessation of breastfeeding and increased consumption of fatty or sugary foods at 1 year of age are risk factors for infection [32].

Majority of the children were given mixed feeding before the ages of 6 months in all the study sites and children who were not given breast milk at all from birth, had the highest prevalence of malnutrition. This is in line with studies by Grummer-Strawn *et al*. [32] and Awogbenja and Ugwuona [31] in Nigeria. The significantly low practice of EBF among parents with no formal education may be attributed to their lack of information on the importance of EBF, and the wrong perceptions mothers have about feeding breast milk alone for the recommended duration of 6 months. Majority of mothers in the study areas perceived that, the baby was not satisfied with breast milk, as such felt the need for early commencement of complementary feeding.

Malnutrition was common (32.6%) in all the three localities. This is lower than the 58.1% observed in children in some parts of the Mount Cameroon area by Nkuo-Akenji *et al*. [33] and higher than the 30.2% reported in Dibanda in the same area by Mbuh and Nembo [34]. Stunting was the most common form of malnutrition with a prevalence of 27.1% which is lower than the 38.64% reported by Tine *et al*. [35] in Senegal, the 42.9% obtained by Akiyama *et al*. [20] in Loa People’s Democratic Republic and 30.0% by Magalhães *et al*. [36] in the Northern part of Angola. However, a lower prevalence of 17.1% was obtained by Sumbele *et al*. [21] in children of Muea community in the same Mount Cameroon area following education on the feeding habits and 19.4% by Nyaaba *et al*. [37] in Ghana. The common occurrence of this condition in communities where majority of the inhabitants are farmers may be due to insufficient energy food and nutrient intake, which comes as a result of the fact that, the parents do not really devote time to feed their children properly and rely mostly on caregivers. Furthermore, the higher prevalence of stunting could also be attributed to the decline in socio- economic activities and the instability in the region. At the time of sampling, most of the inhabitants from neighbouring communities around the Mount Cameroon area had migrated to these study sites. As a consequence, most of the homes were overcrowded and having proper nutrition became a challenge.

The significant decrease in prevalence of malnutrition and its forms with age is similar to findings reported by Marriott *et al*. [38] in the Eastern parts of Cameroon. The trend in decline of prevalence of stunting with age observed in the ≤2 years old (31.8%), the 2.1–4.0 years (30.2%) and the 4.1–5 years (18.2%) age groups, is in line with the national statistics for Cameroon for children 18‐23 months (42.4%), 24‐35 months (42.3%), and 36‐47 months (38.7%) of age [39]. The reasons for the decrease in malnutrition with increase in age may likely be due to the fact that, the children are now able to eat a variety of affordable meals that includes vegetables produced by their parents through small scale farming activities. Children who were EBF had the lowest prevalence of wasting. This is similar to studies reported by Onis *et al*. [40] in Nigeria. This could be attributed to the fact that; breast milk constitutes all the classes of food in their rightful proportions to meet up with the growing energy demand and study growth as well.

The low prevalence of malaria parasitaemia among children with malnutrition is in line with studies from the Democratic Republic of Congo [41]. On the contrary, other studies [42, 43] reported malaria as a risk factor for malnutrition. Findings from the study revealed a significant association between infant feeding practice and the prevalence of malaria parasite, with a significantly lower prevalence in children who were exclusively breastfed. This is similar to a study carried out in Kinshasa, Democratic Republic of the Congo [44]. The lower prevalence of malaria parasite infection among EBF infants may be the result of early development of anti-parasite immunity unlike their counterparts who were not breastfed. This is probably due to the transfer of maternal IgG antibodies against *P*. *falciparum* present in breast milk which is systemically absorbed into blood circulation where malaria parasites reside as well [45, 46].

The association between malaria parasitaemia and anaemia has been reported in previous studies within the Mount Cameroon area [47, 48] and in Rwanda [28]. The occurrence of malarial anaemia cases (23.5%) in children less than 5 years is not an uncommon finding in this part of the country [12] and elsewhere [49]. This supports the role malaria plays in the pathogenesis of anaemia in this community. Moreover, a higher prevalence of anaemia was observed in children of the ≤2 years’ age group which is a period of optimal growth, and includes those on transition from breast milk to complementary feeding hence, they may be more vulnerable to infections including malaria parasites resulting in severe and fatal consequences.

Findings from the study showed that 77.3% of infants’ ≤5 years of age in the Mount Cameroon area were anaemic indicating anaemia is a severe public health problem based on the WHO classification [50]. This high prevalence of anaemia could be attributed to the fact that, majority of the mothers in these study areas are farmers who abandon their children back home at very tender ages. Also, most of their farm products are taken to the market to sell hence, the children are likely not well fed and taken care of. In addition to this, the socio-economic instability in South west Cameroon has affected mostly the farmers in the rural and semi-rural communities. Majority of the farmers have been internally displaced from their farm lands and are seeking refuge in safer localities nearby. Consequently, with the farm produce abandoned in bushes and increase in the number of inhabitants per household, providing good nutrition for the children is a challenge.

Over 45.31% of the infants suffered from moderate anaemia. These results are consistent with results of other studies on anaemia among infants in the same area [51]. The high occurrence of anaemia in males than female is similar to other reports [52] however; this contradicts other studies which found no association between anaemia and gender [53]. The high occurrence of anaemia in boys may be related to the faster growth of pre-school boys compared to girls, which results in a high iron demand that cannot be met by diet alone [53]. However, further studies are necessary to better understand this factor.

The high prevalence of anaemia in children given complementary food before 6 months could be attributed to the fact that, the complementary feed unlike breast milk is not uniquely suited to the infants’ nutritional needs and it is not given in adequate proportions. In 2009, WHO reported that, infant and young child feeding practice is suboptimal throughout the world especially the late initiation of breastfeeding, prelacteal feeding, early or late introduction of optimal complementary foods, giving poor quality, quantity and unhygienic complementary food, and using a bottle to feed the child are the common practices in developing countries [54, 55]. Adequate nutrition during infancy and early childhood is essential to ensure the growth, health and development of children to their full potential [7]. Hence, the first two years of life provide a critical window of opportunity for prevention of growth faltering and under-nutrition through optimal feeding [56].

Findings from the study revealed that breastfeeding exclusively for up to 6 months of age is associated with significantly lower rates of anaemia among the children. The lower rate of anaemia in EBF children is not unusual and highlights the essence of the practice. Breast milk is uniquely suited to infants’ nutritional needs, and contains unparalleled immunological and anti-inflammatory properties that protect against a host of illnesses and diseases [1], unlike formula feeding and early weaning from breast milk that has been linked to higher rates of a number of serious health conditions. Other studies have identified weaning after 6 months as a risk factor for iron deficiency anaemia in resource-limited countries, where infants are more likely to have lower iron stores at birth [57, 58]. Furthermore, this study revealed that, children from single parents were at higher risk of anaemia than children from married parents. This could be attributed to the challenges faced by single parents in raising a family especially the farmers who are of low socio-economic status. The parental care given to the children is minimal as the mother spends more time sourcing for income to cater for the needs of the family as a whole.

## Conclusions

Of great significance is the influence of infant feeding habits on the prevalence of *P*. *falciparum* parasitaemia in the study area, with the highest occurrence in children not breastfed. Hence, the practice of exclusive breastfeeding should be encouraged for up to 6 months in malaria endemic zones to improve on the health and growth of the children. Anaemia is a severe public health problem among children **≤**5 years of age in this area hence, control measures to curb the burden should focus on children **≤**2 years of age, those from single parents and those whose parent had no formal education. Iron fortified diets should be given to infants who are given complementary feeding before the first months of life in order to reduce the prevalence of anaemia within the first six months of age. The study had as limitations some unmeasured factors such as micro-nutrient deficiency, and markers of inflammation which may have acted as confounders on the risk of the presence of anaemia. Never the less, the findings of the study demonstrated the invaluable contribution of infant feeding habits to these public health problems in children ≤5 years of age.

## Supporting information

S1 Manuscript Dataset (XLSX)
